# Post-laparoscopic sleeve gastrectomy changes in circulating magnesium and bone mineral density indices: A 12-month prospective study

**DOI:** 10.1097/MD.0000000000047774

**Published:** 2026-02-20

**Authors:** Safaa A. Alsaaydan, Hanan A. Alfawaz, Mohammed S. Almohaya, Nasreen Alfaris, Ahmad A. Al-Ghamdi, Ali A. Alshehri, Youssef A. Alsuhaibani, Saud D. Alzahrani, Malak Nawaz K. Khattak, Shaun Sabico, Sobhy M. Yakout, Nasser M. Al-Daghri

**Affiliations:** a Department Food Science and Nutrition, King Saud University, Riyadh, Saudi Arabia; b Obesity, Endocrine and Metabolism Center, King Fahad Medical City, Riyadh, Saudi Arabia; c Department of Surgery, King Fahad Medical City, Riyadh, Saudi Arabia; d Chair for Biomarkers of Chronic Diseases, Department of Biochemistry, College of Science, King Saud University, Riyadh, Saudi Arabia.

**Keywords:** BMD, dual-energy X-ray absorptiometry, LSG, magnesium, Saudi Arabia, sleeve gastrectomy

## Abstract

Laparoscopic sleeve gastrectomy (LSG) is one of the most popular weight-loss surgeries globally. Despite its effectiveness in weight loss, LSG may reduce serum magnesium (Mg) levels and impact bone health negatively. The current prospective study determined whether patients experienced changes in serum Mg levels and bone mineral density (BMD) for the total body, spine L1–L4, neck femur, and total hip 12 months post-LSG. A total of 51 (19 males and 32 females) out of 72 patients who underwent LSG were able to complete 12 months of follow-up. Anthropometric measurements, BMD, and serum Mg levels were assessed at baseline, as well as at 3 and 12 months after surgery. Total BMD significantly decreased in all cases by 2.6% in 3 months and by 4.3% in 12 months after surgery (*P*-values < .01). There was a significant increase in serum Mg levels 12 months post-LSG (+0.04 mmol/L; *P* < .01). In all cases, regression analysis revealed that BMD modestly but significantly explained 5% of the variations in serum Mg (β = −1.4 [standard error, SE 0.6]; *P* = .04) 3 months post-LSG. After a year, this perceived variance increased to 9% (β = −0.22 [0.07]; *P* = .006). In conclusion, both serum Mg levels and BMD are significantly altered 12 months following LSG. Whether these changes represent clinically meaningful effects or reflect transient, compensatory physiological adaptations warrants further investigation.

## 1. Introduction

Recent data show that approximately 1 billion people suffer from obesity worldwide.^[1]^ Despite the efforts of countries and international organizations, the number of obesity-related deaths is likely to continue growing in the near future.^[1–4]^ The terms “overweight” and “obese” are typically used based on the clearly established criteria related to body mass index (BMI).^[5–7]^ Researchers have found links between fat and a number of health problems. It is known to aggravate mental health problems,^[7]^ increase the risks of infertility,^[8]^ cause different cancers,^[9]^ lead to joint problems,^[10]^ and increase the risk of developing type 2 diabetes.^[11]^ The high prevalence of obesity is known to increase mortality rates, although the relationship between obesity prevalence and death rates can be mediated by many moderating variables, such as lifestyle behaviors, the quality of the healthcare system, and confounding risk factors.^[4]^ Understanding these factors enhances our comprehension of the full range of health issues related to obesity and the most effective prevention strategies.

Most conventional strategies to reduce weight, such as changing dietary habits and exercising, are effective yet difficult to implement.^[12–15]^ Contrary to them, bariatric surgery (BS) is known to achieve swift and impressive outcomes in treating severe obesity.^[16]^ There are several types of BS and these include gastric bypass, sleeve gastrectomy, adjustable gastric banding, and biliopancreatic diversion with duodenal switch.^[17–20]^ Laparoscopic sleeve gastrectomy (LSG) is the most popular type of BS. It implies creating a sleeve-shaped stomach via the removal of a large part of the stomach.^[21]^ LSG has been associated with a sustainable decrease in BMI,^[22]^ increased average life expectancy,^[23]^ and reduced risks of suffering from dangerous health conditions triggered or aggravated by obesity, such as diabetes, hypertension, and others.^[24,25]^ Moreover, the procedure is considered safe and is associated with significant weight reduction effects.^[21–25]^

Despite the promising benefits of BS, there are concerns that it can adversely affect bone metabolism in the long-term perspective.^[26,27]^ One of the channels through which such an effect can manifest itself is through changes in magnesium (Mg) levels. Mg plays a major role in bone metabolism since it enhances the activity of alkaline phosphatase^[28]^ and assists with building bone tissue.^[29]^ A set of studies indicate that low Mg levels contribute to low bone density and high bone resorption,^[30–32]^ while Mg supplements are given to patients post-LSG with Mg deficiency.^[33–35]^

The existing evidence for changes in Mg levels in LSG patients is fragmentary and inconsistent. In one of the studies, the percentage of patients with Mg deficiency increased from 70.33% presurgery to 93.40% in a 1-year follow-up.^[32]^ In another study, the percentage of individuals with low Mg levels fell from 37.8% to 12.5% in 60 months after the surgery.^[31]^ Similar findings also were reported in 2 other studies.^[30,36]^ Despite the fragmentary nature of scientific evidence for the impact of LSG on Mg levels, health specialists often recommend LSG patients take Mg supplements.^[34,35]^ In general, changes in Mg levels reported by LSG patients remain poorly understood. Some authors doubt that LSG contributes to low Mg levels,^[30,31,36–38]^ while others believe that a possible risk of developing Mg deficiency has already become a critical concern within the context of LSG.^[32,34,35]^ In theory, LSG may predispose patients to Mg deficiency through reduced dietary intake, diminished gastric acid–mediated mineral solubilization, and altered gastrointestinal transit that can impair absorption despite preserved intestinal anatomy.^[34,35]^ In addition, postoperative metabolic and hormonal changes, rapid weight loss, and suboptimal supplementation may increase renal Mg losses and lower circulating levels.^[36–38]^ The current study seeks to address this evident research gap by producing valid scientific evidence for the changes in serum Mg levels of LSG patients and their impact on bone density.

## 2. Materials and methods

### 2.1. Study design and patients

**Figure 1.**
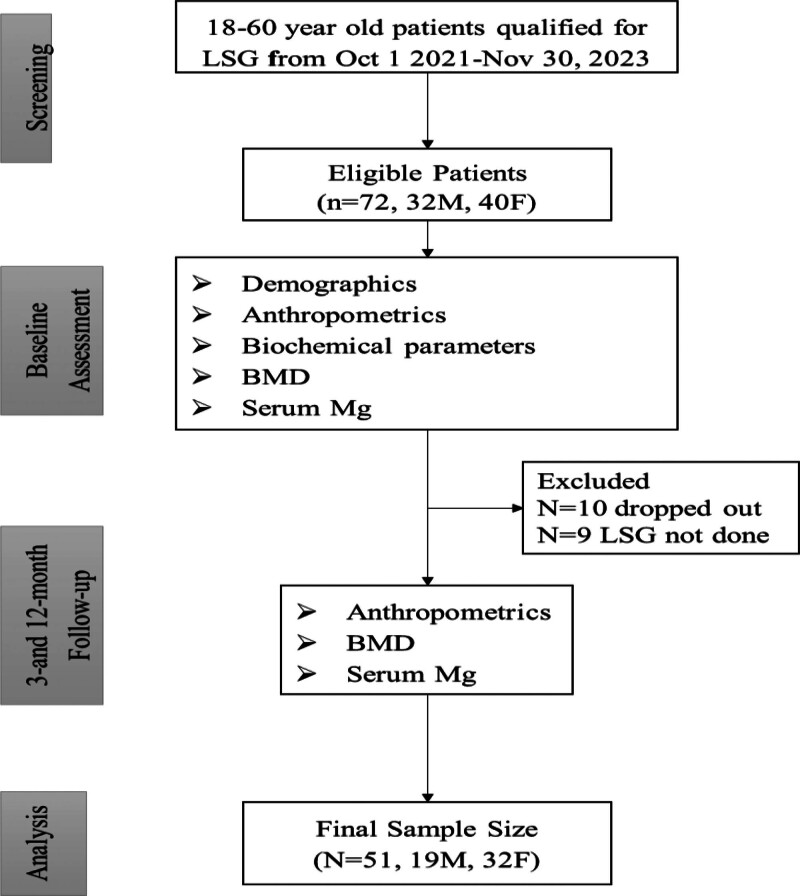
Flowchart of patients. BMD = bone mineral density, LSG = laparoscopic sleeve gastrectomy.

The present prospective, single-center cohort study was carried out at King Fahad Medical City (KFMC) in Riyadh, Saudi Arabia and followed the Strengthening the Reporting of Observational Studies in Epidemiology statement for cohort studies. The individuals had to meet a set of inclusion criteria, including a high BMI justifying LSG (at least 40 kg/m^2^ or at least 35 kg/m^2^ and the underlying comorbidities), being aged between 18 and 60 years old, and the absence of previous bone disease history. All female patients were premenopausal at baseline. All patients enrolled in the study signed an informed consent form. Participants were excluded if they were outside the eligible age or BMI criteria, had undergone prior bariatric or major upper gastrointestinal surgery, were postmenopausal, pregnant or lactating, had known metabolic or chronic bone disease, chronic kidney or significant liver disease, endocrine disorders or chronic conditions affecting bone metabolism, were using medications known to influence bone turnover, or were unable or unwilling to complete follow-up assessments. The study was approved by the Research Ethics Department Faculty of Medicine in KFMC Research Center (IRB No. 21-005). A total of 72 patients underwent LSG from October 1, 2021, to November 30, 2022 (32 males and 40 females) with baseline data, of whom 51 (19 males and 32 females) were able to complete the full 12-month assessment (Fig. 1).

### 2.2. Surgery

The sleeve gastrectomy was a surgical procedure that the patients underwent in accordance with the appropriate medical recommendations. This procedure involved the removal of a substantial segment of the stomach, resulting in a banana-shaped organ.^[21]^ The technique involved removing approximately 80% of the stomach in each patient. Gastric division was initiated 3 to 4 cm proximal to the pylorus and continued along the corpus toward the angle of His.^[39]^ The next stages of the procedure involved placing a 38-F bougie inside the gastric lumen and leaving a pouch of 80 to 100 mL.^[40]^ The surgeries were successful, as the patients did not suffer from any side effects and did not face any health threats associated with the procedure.

### 2.3. Determinations and medical procedures

Before and after surgery, the following evaluations were carried out: anthropometry, laboratory determinations, and bone mineral density (BMD) measurement. Multivitamin and mineral contents (calcium carbonate 200 mg and Mg 175 mg, 1 tablet/d), vitamin D (50,000 IU/weekly), and vitamin B complex (1 tablet/d) were prescribed for patients after LSG as oral supplements.

### 2.4. Anthropometric evaluations

Height and weight were determined under standard conditions (fasting state, light clothes without shoes) using a medical scale SECA 763 (Seca Gmbh & Co. kg., Hamburg, Germany). The BMI was calculated as a ratio of weight and height squared (kg/m^2^) and evaluated according to the criteria of the World Health Organization for obesity.^[1]^ Waist and hip circumferences were collected before the operation and 3 and 12 months after the operation by using tape.^[41]^

### 2.5. Bone densitometry

Dual-energy X-ray Absorptiometry scans were performed using the GE Lunar Prodigy Advance and GE Encore software version 7 (GE Healthcare, Madison) the National Health and Nutrition Examination Survey reference database was used.^[42]^ BMD measurements of the total body, spine L1–L4, total hip, and neck femur were obtained. We followed the criteria of the World Health Organization for categorizing patients as normal for T-scores above −1 standard deviation.^[42]^ T-scores above −1.0 were considered normal figures, while T-scores below −2.5 and between −1.0 and −2.5 were regarded as possible signs of osteoporosis and osteopenia, respectively, for men over the age of 50 years.^[43]^ Z-score values were applied for premenopausal women and men below the age of 50 years. The study’s main dependent variable was total body BMD, which was used to compare pre- and post-LSG surgery values in g/cm^2^.

### 2.6. Laboratory determinations

Fasting blood samples were collected and analyzed in the laboratory of KFMC at baseline and the 3rd and 12th months postoperative for serum Mg and baseline parathyroid hormone levels using commercially available assays. Baseline fasting glucose, lipid profile, and other minerals (calcium, potassium, sodium, and phosphate) were assessed using a routine chemical analyzer. Serum vitamin D was measured using electrochemiluminescence assays.

### 2.7. Statistical analysis and sample size calculation

Data was analyzed using SPSS version 22.0. (IBM SPSS, Chicago) Frequencies were presented as N (%) while continuous variables were presented as mean ± standard deviation. All continuous variables were checked for normality using the Kolmogorov–Smirnov and the Shapiro–Wilk tests. An independent *T*-test was used to compare differences between male and female subjects. Repeated measures analysis of variance was used to determine changes between variables of interest overtime. Regression analysis was used to determine the relationship between changes in Mg levels and variances in patients’ BMD. Significance was set at *P* < .05.

Sample size determination was calculated using G*Power based on literature on BMD changes. Given the small effect size of 0.49, margin of error = 0.05 and power of the test = 0.95 the required sample size is n = 36.^[44]^

## 3. Results

### 3.1. Baseline characteristics of patients

Table S1, Supplemental Digital Content, https://links.lww.com/MD/R426 presents the full demographic characteristics of patients. Table 1 shows the clinical characteristics of the 51 (19 males and 32 females) patients included in the final analysis. In the anthropometrics, male patients were significantly heavier (*P* < .001) and had larger waists and waist-to-hip ratio (*P*-values < .001 and .02, respectively) than female patients. Females had significantly lower total body BMD (*P* = .007) than males as well as BMD of the femoral neck (*P* = .04). Females also had significantly higher high-density lipoprotein-cholesterol than males (*P* = .006). Age, baseline BMI, and serum Mg, were not different between males and females.

**Table 1:** Clinical characteristics of subjects.

Parameters	All	Males	Females	*P*-value
N	51	19	32	
Age (yr)	35.3 ± 9.1	34.8 ± 12.7	35.6 ± 6.3	.39
BMI (kg/m^2^)	46.8 ± 7.3	47.7 ± 8.1	46.4 ± 6.8	.27
Weight (kg)	127.8 ± 24.2	141.3 ± 28.8	119.7 ± 16.7	<.001
Waist (cm)	128.3 ± 17.6	138.9 ± 15.3	121.9 ± 15.9	<.001
Hips (cm)	135.1 ± 15.1	140.3 ± 17.2	132.1 ± 13.1	.06
WHR	0.95 ± 0.11	0.99 ± 0.08	0.93 ± 0.11	.024
Systolic BP (mm Hg)	130.9 ± 12.6	132.2 ± 11.4	130.2 ± 13.4	.30
Diastolic BP (mm Hg)	78.9 ± 10.5	80.2 ± 10.6	78.1 ± 10.5	.25
Spine BMD L1–L4	1.2 ± 0.14	1.2 ± 0.15	1.2 ± 0.13	.78
T-Score	0.02 ± 1.1	0.15 ± 1.3	−0.05 ± 1.1	.55
z-Score	−0.04 ± 1.1	−0.07 ± 1.2	−0.02 ± 1.1	.44
BMD femoral neck	1.06 ± 0.13	1.11 ± 0.11	1.04 ± 0.13	.042
T-Score	0.14 ± 0.9	0.62 ± 0.97	−0.12 ± 0.85	.04
z-Score	0.47 ± 1.0	0.47 ± 0.73	0.47 ± 0.86	.99
Total body BMD	1.3 ± 0.11	1.33 ± 0.11	1.25 ± 0.10	.007
T-Score	1.5 ± 1.13	1.4 ± 1.2	1.61 ± 1.1	.53
z-Score	1.4 ± 1.16	1.4 ± 1.2	1.61 ± 1.1	.44
Triglycerides (mmol/L)	1.5 ± 1.1	1.7 ± 1.5	1.21 ± 0.4	.08
Total cholesterol (mmol/L)	4.6 ± 0.8	4.62 ± 0.9	4.54 ± 0.8	.74
HDL cholesterol (mmol/L)	1.14 ± 0.23	1.03 ± 0.2	1.20 ± 0.24	.006
LDL cholesterol (mmol/L)	3.3 ± 0.9	3.5 ± 0.9	3.2 ± 0.8	.28
Magnesium (mmol/L)	0.79 ± 0.06	0.81 ± 0.06	0.78 ± 0.06	.06
Potassium (mmol/L)	3.93 ± 0.34	4.1 ± 0.5	3.85 ± 0.2	.04
Calcium (mmol/L)	2.36 ± 0.10	2.4 ± 0.08	2.33 ± 0.1	.74
Phosphate (mmol/L)	1.15 ± 0.3	1.23 ± 0.3	1.10 ± 0.2	.10
Sodium (mmol/L)	137.9 ± 1.9	138.1 ± 2.02	137.8 ± 1.8	.60
Glucose-fasting (mmol/L)	5.90 ± 1.4	6.20 ± 1.9	5.73 ± 1.1	.30
HbA1c (%)	5.94 ± 0.9	6.05 ± 1.0	5.87 ± 1.0	.53
Vitamin D (nmol/L)	55.7 ± 25.6	50.0 ± 22.7	59.1 ± 26.9	.22
PTH	12.5 ± 5.1	11.3 ± 4.8	13.2 ± 5.2	.19

Data presented as mean ± SD and median (IQR); significant at *P* < .05.

BMD = bone mineral density, BMI = body mass index, BP = blood pressure, HbA1c = glycated hemoglobin, HDL = high density lipoprotein, LDL = low density lipoprotein, PTH = parathyroid hormone, SD = standard deviation, WHR = waist-to-hip ratio.

### 3.2. Post-surgery changes overtime

**Figure 2.**
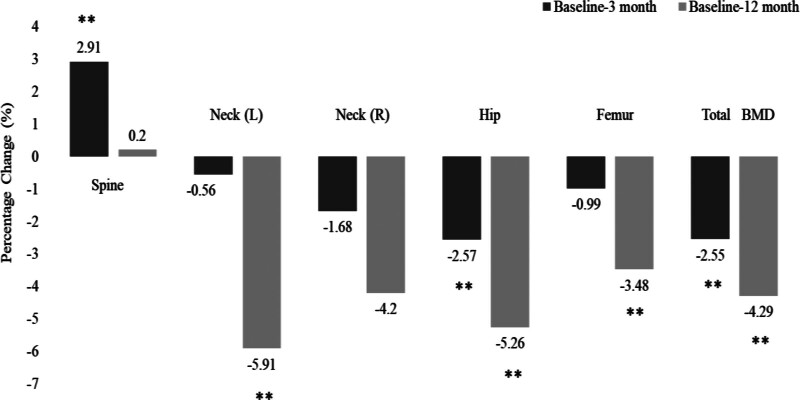
Percent (%) change in BMD Indices in all patients. BMD = bone mineral density.

**Figure 3.**
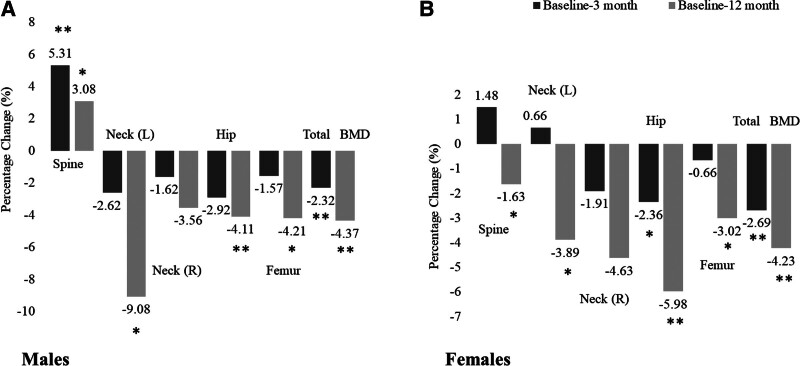
Percent (%) change in BMD indices in (A) males and (B) females. BMD = bone mineral density.

**Figure 4.**
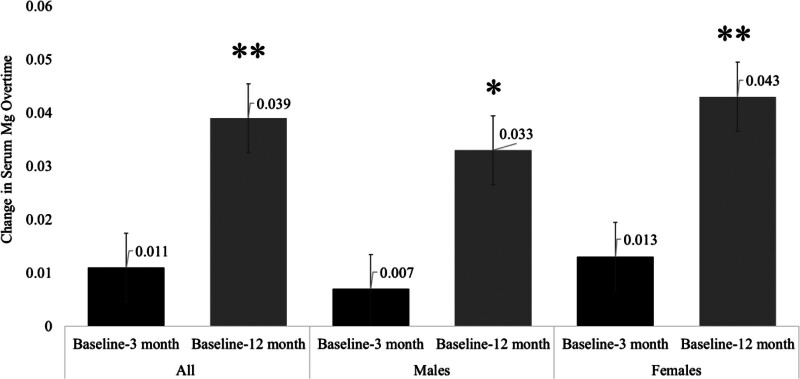
Post-surgery changes in serum Mg in all patients. Mg = magnesium.

Table 2 shows the post-surgery changes in anthropometrics and BMD indices after 3 and 12 months. As expected, there was a significant decrease in all anthropometric indices in all patients. This significance persisted even after stratification according to sex. In terms of changes of BMD, a significant decrease in total hip and total body BMD values was observed at both time points, particularly after 12 months. The follow-up of L1–L4 spine BMD showed a slight increase in males after 3 and 12 months (*P* < .01) and stable L1–L4 BMD values in females. Total BMD significantly decreased in both males and females after 3- and 12-months post-surgery (Table 2). The percentage of changes in BMD indices in all patients as well as in males and females was plotted as Figures 2 and 3. Finally, Figure 4 shows the post-surgery changes in circulating Mg in all patients that showed a significant increase only after 12 months (*P* < .01). This significance was observed in both males and females.

**Table 2:** Mean changes in anthropometrics and BMD indices overtime.

Parameters	All subjects (N = 51)		Males (N = 19)		Females (N = 32)	
	3 months	12 months	3 months	12 months	3 months	12 months
Weight (kg)	−26.0 ± 8.8**	−42.0 ± 14.7**	−30.2 ± 9.5**	−51 ± 15.2**	−23.5 ± 7.5**	−36.7 ± 11.7**
BMI (kg/m^2^)	−9.3 ± 3.1**	−15.0 ± 5.2**	−10.2 ± 2.9**	−20.9 ± 8.8**	−8.8 ± 3.1**	−14.0 ± 5.2**
Waist (cm)	−16.4 ± 12.3**	−30.1 ± 16.7**	−20.9 ± 8.8**	−37.8 ± 8.7**	−13.7 ± 13.4**	−25.5 ± 18.7**
Hips (cm)	−14.5 ± 11.3**	−25.7 ± 14.9**	−18.3 ± 9.5**	−31.1 ± 12.1**	−12.2 ± 11.9**	−22.3 ± 15.7**
*BMD*						
L1–L4 spine	0.04 ± 0.06**	0.003 ± 0.07	0.06 ± 0.05**	0.04 ± 0.06*	0.02 ± 0.06	−0.02 ± 0.06
F-neck (L)	−0.008 ± 0.06	−0.06 ± 0.12**	−0.03 ± 0.06	−0.09 ± 0.16*	0.006 ± 0.06	−0.04 ± 0.09*
F-neck (R)	−0.06 ± 0.12	−0.05 ± 0.15*	−0.02 ± 0.09	−0.04 ± 0.08	−0.02 ± 0.12	−0.05 ± 0.19
Total hip	−0.03 ± 0.06**	−0.06 ± 0.08**	−0.04 ± 0.03	−0.05 ± 0.11**	−0.03 ± 0.07*	−0.07 ± 0.07**
Neck femur	−0.01 ± 0.08	−0.04 ± 0.07**	−0.02 ± 0.08	−0.04 ± 0.08*	−0.008 ± 0.08	−0.04 ± 0.07*
Total BMD	−0.03 ± 0.05**	−0.06 ± 0.06**	−0.03 ± 0.04**	−0.06 ± 0.05**	−0.03 ± 0.05**	−0.05 ± 0.06**

Data presented as mean ± SD and median (IQR).

BMD = bone mineral density, BMI = body mass index, F = femoral, L = left, R = right, SD = standard deviation.

* Denotes significance at .05 level.

** Denotes significance at .01 level.

### 3.3. Associations of BMD to variances perceived in Mg levels overtime

Linear regression was performed using circulating Mg level as the dependent variable and the different BMD indices as independent variables (spine, femur [R, L, neck], hip, and total body) and presented as in Table S1, Supplemental Digital Content, https://links.lww.com/MD/R426. In all patients, BMD modestly but significantly explains 5% of the variations in serum Mg (β = −1.4 [standard error, SE 0.6]; *P* = .04) 3 months post-surgery. This perceived variance increases to 9% after 12 months (β = −0.22 [0.07]; *P* = .006). Stratification according to sex revealed no association between BMD and serum Mg in males. In females, however, BMD explained 6% of the perceived variance in serum Mg 3 months post-surgery [β = −1.4 (0.7); *P* = .047] and 4% after 12 months (β = −0.25 [0.1]; *P* = .03). The same regression analysis was performed stratified according to age and revealed that for patients <30 years old, BMD explained 37% of the variances perceived in serum Mg at presurgery (β = −0.75 [0.25]; *P* = .02). This significance was lost after 3 months, and a 12-month follow-up was done. For individuals 30 to 40 years old as well as 40 years and above, no association was found between BMD at baseline and 3-month follow-up. However, after 12 months, BMD was significantly associated with serum Mg by 31% and 54%, respectively (*P*-values .04 and .01, respectively)

## 4. Discussion

The present 12-month study investigated the changes in circulating Mg and BMD indices among severely obese Saudi patients who underwent LSG and found that serum Mg levels significantly increased overtime in 12 months with a parallel significant decrease in total BMD in both males and females. Furthermore, the changes in the levels of Mg observed were significantly associated, albeit in varying degrees, with the decrease in BMD indices, and these variations were substantially influenced by sex and age. The findings contradict a recent study suggesting that LSG increases the risk of Mg deficiency 1 year following operation.^[32]^ The study also did not find confirmation of the need to recommend LSG patients take Mg^2+^ supplements in order to prevent Mg deficiency.^35,36^ The study demonstrated a decrease in the number of patients with low Mg levels 1 year after LSG, something that is in line with several other studies examining the implications of LSG.^[30,31,36]^ Therefore, the study confirmed the existence of a link between LSG and serum Mg levels but showed that the surgery generally leads to higher Mg levels.

There are several potential explanations for the increase in Mg levels in LSG patients. In particular, the patients could have benefited from enhanced nutrient absorption after the surgery.^[21,22]^ Another possible factor is that changes in body composition could have affected Mg metabolism. It was found that LSG patients experienced a decline in their BMI, weight, total lean mass, total fat mass, WHT, neck circumference, and waist. These changes may explain the increase in Mg levels.

The results of this research indicate that LSG patients might experience a decline in their BMD for the total body, neck femur, spine L1–L4, and total hip. At the same time, it is important to point out that the fall in all these indices is slight; furthermore, some of them are not statistically significant within both 3 and 12 months after the surgery. The values of BMD are not indicative of significant problems with bone health and do not present a risk of osteoporosis in the short term. It should be noted, however, that the sample did not include patients who suffered from osteoporosis. As a result, it does not seem possible to analyze the impact of LSG on individuals with poor bone health in this study.

In general, the findings of the research conform to the existing knowledge of the link between LSG and bone density.^[26,27]^ At the same time, a decline in bone density reported in this study could not be attributed to changes in Mg levels as suggested in previous research^[30–32]^ Apparently, it was a result of other factors, such as changes in body composition and malabsorption.

The study found that LSG could trigger changes in serum Mg levels and BMD. An increase in Mg levels 12 months post LSG was observed. Furthermore, BMD values of total body and total hip declined significantly both 3 and 12 months after the surveys. An increase in serum Mg levels could not predict the variance of the patients’ BMD based on the data from 12-month follow-up measurements. The current study conforms to the existing knowledge of the link between LSG and bone health. It also highlights the findings reported concerning the positive impact of LSG on Mg levels. These data do not confirm the need for specific Mg supplements for LSG patients. Moreover, the study did not demonstrate an accelerated bone loss post-LSG surgery since the decline in BMD was slight. These findings are subject to several limitations, such as a relatively small sample size and the lack of detailed information about patients’ diets after the survey. We must take these limitations into account when applying the study’s findings in practice or conducting additional research.

Further research is needed to better understand the changes in Mg levels in LSG patients. Scientists might consider analyzing possible reasons behind an increase in serum Mg levels attributed to LSG, such as dietary adjustments, changes in body composition, and malabsorption. It also might be useful to address other variables that contribute to BMD changes and the impact of LSG surgeries on fracture risk.

## 5. Conclusion

In this 12-month prospective cohort of Saudi adults undergoing LSG, circulating Mg exhibited a modest increase at 1 year, while total body and total hip BMD demonstrated a slight decline at 3 and 12 months, with lumbar spine values remaining largely stable overall, showing a slight increase in males. Alterations in Mg accounted for merely a minor fraction of the variance in BMD and exhibited patterns specific to sex and age, indicating that post-LSG bone loss is improbable to be solely influenced by Mg. The findings advocate for the regular assessment of BMD and mineral status post-LSG, the supplementation of vitamin D and calcium (± Mg if deficient), and the implementation of lifestyle modifications that safeguard bone health, including sufficient protein intake and weight-bearing or resistance exercise. More extensive, longitudinal studies that incorporate dietary factors are necessary to elucidate mechanisms and fracture risk trajectories beyond the initial postoperative year.

## Acknowledgments

The authors are thankful to the Ongoing Research Funding Program-Research Chairs (ORF-RC-2026-1400), King Saud University, Riyadh, Saudi Arabia, for funding this research.

## Author contributions

**Conceptualization:** Safaa A. Alsaaydan, Hanan A. Alfawaz, Mohammed S. Almohaya.

**Data curation:** Nasreen Alfaris, Ahmad A. Al-Ghamdi, Ali A. Alshehri, Youssef A. Alsuhaibani, Saud D. Alzahrani.

**Formal analysis:** Malak Nawaz K. Khattak.

**Funding acquisition:** Nasser M. Al-Daghri.

**Investigation:** Safaa A. Alsaaydan, Mohammed S. Almohaya, Nasreen Alfaris, Ahmad A. Al-Ghamdi, Ali A. Alshehri, Youssef A. Alsuhaibani, Saud D. Alzahrani.

**Methodology:** Safaa A. Alsaaydan, Mohammed S. Almohaya, Nasreen Alfaris, Ahmad A. Al-Ghamdi, Ali A. Alshehri, Youssef A. Alsuhaibani, Saud D. Alzahrani.

**Project administration:** Sobhy M. Yakout.

**Supervision:** Hanan A. Alfawaz, Shaun Sabico, Nasser M. Al-Daghri.

**Writing – original draft:** Safaa A. Alsaaydan, Shaun Sabico, Sobhy M. Yakout.

**Writing – review & editing:** Hanan A. Alfawaz, Mohammed S. Almohaya, Nasreen Alfaris, Ahmad A. Al-Ghamdi, Ali A. Alshehri, Youssef A. Alsuhaibani, Saud D. Alzahrani, Malak Nawaz K. Khattak, Sobhy M. Yakout, Nasser M. Al-Daghri.

## Supplementary Material

## References

[1] World Health Organization. World obesity day 2022- accelerating action to stop obesity. 2022. WHO. Available at: https://www.who.int/news/item/04-03-2022-world-obesity-day-2022-accelerating-action-to-stop-obesity. Accessed November 3, 2023.

[2] Nianogo RA Arah OA. Forecasting obesity and type 2 Diabetes incident and burden: the VilA – obesity simulation model. Front Public Health. 2022;10:818816. 35450123 10.3389/fpubh.2022.818816 PMC9016163

[3] Ward ZJ Bleich SN Cradock AL. Projected U.S. state-level prevalence of adult obesity and severe obesity. N Engl J Med. 2019;381:2440–50. 31851800 10.1056/NEJMsa1909301

[4] Ritchie H Roser M. Obesity. Our World in Data. 2020. Available at: https://ourworldindata.org/obesity. Accessed November 3, 2023.

[5] Tate CM Geliebter A. Intragastric balloon treatment for obesity: review of recent studies. Adv Ther. 2017;34:1859–75. 28707286 10.1007/s12325-017-0562-3

[6] Rawlins L Rawlins MP Brown CC Schumacher DL. Sleeve gastrectomy: 5-year outcomes of a single institution. Surg Obes Relat Dis. 2012;9:21–5. 23201209 10.1016/j.soard.2012.08.014

[7] Small L Aplasca A. Child obesity and mental health: a complex interaction. Child Adolesc Psychiatr Clin N Am. 2016;25:269–82. 26980129 10.1016/j.chc.2015.11.008

[8] Marinelli S Napoletano G Straccamore M Basile G. The impact of female obesity on fertility capabilities and on future pregnancy prospects: a mini-review. Acta Biomed. 2022;93:1–13.

[9] Lauby-Secretan B Scoccianti C Loomis D. Body fatness and cancer – viewpoint of the IARC working group. N Engl J Med. 2016;375:794–8. 27557308 10.1056/NEJMsr1606602 PMC6754861

[10] Gudbergsen H Boesen M Christensen R. Changes in bone marrow lesions in response to weight-loss in obese knee osteoarthritis patients: a prospective cohort study. BMC Musculoskelet Disord. 2013;14:1–19. 23522337 10.1186/1471-2474-14-106 PMC3618315

[11] Lee H Shin H Oh J. Association between body mass index and outcomes in patients with return of spontaneous circulation after out-of-hospital cardiac arrest: a systematic review and meta-analysis. Int J Environ Res Public Health. 2021;18:8389. 34444142 10.3390/ijerph18168389 PMC8394455

[12] Semlitsch T Krenn C Jeitler K Berghold A Horvath K Siebenhofer A. Long-term effects of weight-reducing diets in people with hypertension. Cochrane Database Syst Rev. 2021;2021:1–15. 10.1002/14651858.CD008274.pub4 PMC8093137 33555049

[13] Jackson SE Llewellyn CH Smith L. The obesity epidemic – nature via nurture: a narrative review of high-income countries. Sage Open Med. 2020;8:1–11. 10.1177/2050312120918265 PMC7222649 32435480

[14] Hall KD Kahan S. Maintenance of lost weight and long-term management of obesity. Med Clin North Am. 2018;102:183–97. 29156185 10.1016/j.mcna.2017.08.012 PMC5764193

[15] Monnier L Schlienger JJ Colette C Bonnett F. The obesity treatment dilemma: Why dieting is both the answer and the problem? A mechanistic overview. Diabet Metab. 2021;47:101192. 10.1016/j.diabet.2020.09.002 33002604

[16] Ozsoy Z Demir E. Which bariatric procedure is the most popular in the world? A bibliometric comparison. Obes Surg. 2018;28:2339–52. 29512038 10.1007/s11695-018-3163-6

[17] Oppenländer L Palit S Stemmer K. Vertical sleeve gastrectomy triggers fast β-cell recovery upon overt diabetes. Mol Metab. 2021;54:101330. 34500108 10.1016/j.molmet.2021.101330 PMC8487975

[18] Poublon N Chidi I Bethelem M. One anastomosis gastric bypass vs. Roux-en-Y gastric bypass, remedy for insufficient weight loss and weight regain after failed restrictive bariatric surgery. Obes Surg. 2020;30:3287–94. 32307669 10.1007/s11695-020-04536-x PMC7378100

[19] Rao R Mehta M Sheth DR Hogan G. Four-year nutritional outcomes in single-anastomosis duodenal-ileal bypass with sleeve gastrectomy patients: an Australian experience. Obes Surg. 2023;33:750–60. 36698049 10.1007/s11695-023-06461-1 PMC9877492

[20] Salet JA Marchesini JB Paiva DS. Brazilian multicenter study of the intragastric balloon. Obes Surg. 2004;14:991–8. 15329191 10.1381/0960892041719671

[21] Nguyen NT Varela JE. Bariatric surgery for obesity and metabolic disorders: state of the art. Nat Rev Gastroenterol Hepatol. 2017;14:160–9. 27899816 10.1038/nrgastro.2016.170

[22] Bartosiak K Rozanska-Waledziak A Waledziak M Kowalewski P Pasnik K Janik MR. The safety and benefits of laparoscopic sleeve gastrectomy in elderly patients: a case-control study. Obes Surg. 2019;29:2233–7. 31020498 10.1007/s11695-019-03830-7

[23] Nevo N Eldar SM Lessing Y Sabo E Nachmany I Hazzan D. Sleeve gastrectomy in the elderly. Obesity Facts. 2019;12:502–8. 31610540 10.1159/000502697 PMC6876611

[24] Jaunoo SS Southall PJ. Bariatric surgery. Int J Surg. 2010;8:86–9. 20026002 10.1016/j.ijsu.2009.12.003

[25] Arterburn DE Telem DA Kushner RF Courcoulas AP. Benefits and risks of bariatric surgery in adults: a review. JAMA. 2020;324:879–87. 32870301 10.1001/jama.2020.12567

[26] Aaseth JO Alexander J. Postoperative osteoporosis in subjects with morbid obesity undergoing bariatric surgery with gastric bypass or sleeve gastrectomy. Nutrients. 2023;15:1302–11. 36986032 10.3390/nu15061302 PMC10057453

[27] Tian Z Fan XT Li SZ Zhai T Dong J. Changes in bone metabolism after sleeve gastrectomy versus gastric bypass: a meta-analysis. Obes Surg. 2020;30:77–86. 31414297 10.1007/s11695-019-04119-5

[28] Mederle OA Balas M Ioanoviciu SD Gurban CV Tudor A Borza C. Correlations between bone turnover markers, serum magnesium, and bone mass density in postmenopausal osteoporosis. Clin Interv Aging. 2018;13:1383–9. 30122910 10.2147/CIA.S170111 PMC6080660

[29] Mammoli F Castiglioni S Parenti S. Magnesium is a key regulator of the balance between osteoclast and osteoblast differentiation in the presence of Vitamin D_3_. Int J Mol Sci . 2019;20:385–94. 30658432 10.3390/ijms20020385 PMC6358963

[30] Heusschen L Schijns W Ploeger N. The true story on deficiencies after sleeve gastrectomy: results of a double-blind RCT. Obes Surg. 2020;30:1280–90. 31776782 10.1007/s11695-019-04252-1

[31] Moize V Andreu A Flores L. Long-term dietary intake and nutritional deficiencies following sleeve gastrectomy or Roux-En-Y gastric bypass in a Mediterranean population. J Acad Nutr Dietetics. 2013;113:400–10. 10.1016/j.jand.2012.11.013 23438491

[32] Mulita F Lampropoulos C Kehagias D. Long-term nutritional deficiencies following sleeve gastrectomy: A 6-year single-center retrospective study. Prz Menopauzalny. 2021;20:170–6. 35069068 10.5114/pm.2021.110954 PMC8764960

[33] Arnett TR. Extracellular pH regulates bone cell function. J Nutr. 2008;138:415S–8S. 18203913 10.1093/jn/138.2.415S

[34] Jastrzebska-Mierzynska M Ostrowska L Witczak-Sawczuk K Hady HR. Assessment of the clinical condition and way of patients’ nutrition before and after laparoscopic sleeve gastrectomy. Nutrients. 2023;15:1–24. 10.3390/nu15030514 PMC9919987 36771221

[35] Wawrzyniak A Krotki M. The need and safety of mineral supplementation in adults with obesity post bariatric surgery – sleeve gastrectomy (SG). Obes Surg. 2021;31:4502–10. 34345960 10.1007/s11695-021-05639-9 PMC8458182

[36] Guan B Yang J Chen Y Yang W Wang C. Nutritional deficiencies in Chinese patients undergoing gastric bypass and sleeve gastrectomy: prevalence and Predictors. Obes Surg. 2018;28:2727–36. 29754386 10.1007/s11695-018-3225-9

[37] Emile SH Elfeki H. Nutritional deficiency after sleeve gastrectomy: a comprehensive literature review. Gastroenterology. 2017;1:99–105.

[38] Timofte D Ochiuz L Ursaru M. The biochemical effect of laparoscopic sleeve gastrectomy on serum magnesium levels. Rev Chim. 2017;68:1997–2001.

[39] Manuel R Fernanndo C Pamela R. Heme- and nonheme-iron absorption and iron status 12 mo after sleeve gastrectomy and Roux-en-Y gastric bypass in morbidly obese women. Am J Clin Nutr. 2012;96:810–7. 22952172 10.3945/ajcn.112.039255

[40] Braghetto I Korn O Valladares H. Laparoscopic sleeve gastrectomy: surgical technique, indications, and clinical results. Obes Surg. 2007;17:1442–50. 18219770 10.1007/s11695-008-9421-2

[41] Kramer JR Fischmach LA Richardson P. Waist-to-hip ratio, but not body mass index, is associated with an increased risk of Barrett’s esophagus in white men. Clin Gastroenterol Heapotol. 2013;11:373–81. 10.1016/j.cgh.2012.11.028 PMC3606681 23220167

[42] Centers for Disease Control and Prevention. National health and nutrition examination survey. CDC. Available at: https://www.cdc.gov/nchs/nhanes/. Accessed February 11, 2026.

[43] Carey JJ Delaney MF Love TE. Dual-energy x-ray absorptiometry diagnostic discordance between z-scores and t-scores in young adults. J Clin Densitometry. 2009;12:11–6. 10.1016/j.jocd.2008.11.001 19195620

[44] Ivaska KK Huovinen V Soinio M. Changes in bone metabolism after bariatric surgery by gastric bypass or sleeve gastrectomy. Bone. 2017;95:47–54. 27818311 10.1016/j.bone.2016.11.001

