# Supplementary material for: Post-laparoscopic sleeve gastrectomy changes in circulating magnesium and bone mineral density indices: A 12-month prospective study
Source: Medicine (Baltimore). 2026 Feb 20;105(8):e47774. doi: 10.1097/MD.0000000000047774 (PMC12928880; doi:10.1097/MD.0000000000047774)
Supplement: Supplementary file 1 [file medi-105-e47774-s001.docx]

**Table S1**: Baseline Demographic Characteristics (Data presented as N (%), significant at p<0.05.)

| Parameters | All | Males | Females | P-value |
| --- | --- | --- | --- | --- |
| N(M/F) | 72 | 32 | 40 |  |
| **Marital Status**  Single  Married  Widow  Divorce | 25 (34.7)  39 (54.2)  2 (2.8)  6 (8.3) | 14 (43.8)  17 (53.1)  1 (3.1)  -- | 11 (27.5)  22 (55.0)  1 (2.5)  6 (15.0) | 0.19 |
| **Education**  Illiterate  Primary  Secondary  University  Postgraduate | 1 (1.4)  12 (16.7)  22 (30.6)  34 (47.2)  3 (4.2) | 5 (15.6)  --  9 (28.1)  15 (46.9)  3 (9.4) | 1 (2.5)  7 (17.5)  13 (32.5)  19 (47.5)  -- | 0.32 |
| **Occupation**  Unemployed  Teacher  Student  Retired  Office work  Medical staff  Housekeeping  Engineer  Other | 10 (13.9)  3 (4.2)  8 (11.1)  8 (11.1)  19 (26.4)  3 (4.2)  16 (22.2)  1 (1.4)  4 (5.6) | 2 (6.3)  1 (3.1)  4 (12.5)  8 (25.0)  13 (40.6)  --  13 (40.6)  1 (3.1)  3 (9.4) | 8 (20.0)  2 (5.0)  4 (10.0)  0  6 (15.0)  3 (7.5)  16 (40.0)  --  1 (2.5) | <0.001 |
| **Medical History**  Food Allergies  Food Intolerance  Constipation  Eating Disorder  Digestive Issues  High Blood Pressure  Joint/back/tendon/muscular  Hypoglycemia  Thyroid problems  Depression/anxiety  Cancer  Low iron/anemia  Lung disease/asthma  Gastroesophageal Reflux  Fatigue/sleep apnea  High Cholesterol  Osteoporosis  Heart Disease  Diabetes | 8 (11.1)  2 (2.8)  26 (36.1)  37 (51.4)  29 (40.3)  25 (34.7)  26 (36.1)  8 (11.1)  11 (15.3)  23 (31.9)  5 (6.9)  10 (13.9)  25 (34.7)  31 (43.1)  37 (51.4)  17 (23.6)  3 (4.2)  11 (15.3)  17 (23.6) | 4 (12.5)  1 (3.1)  13 (40.6)  21 (65.6)  12 (37.5)  11 (34.4)  11 (34.4)  3 (9.4)  1 (3.1)  4 (12.5)  1 (3.1)  --  10 (31.3)  13 (40.6)  16 (50.0)  9 (28.1)  1 (3.1)  6 (18.8)  8 (25.0) | 4 (10.0)  1 (2.5)  13 (32.5)  16 (40.0)  17 (42.5)  14 (35.0)  15 (37.5)  5 (12.5)  10 (25.0)  19 (47.5)  4 (10.0)  10 (25.0)  15 (37.5)  18 (45.0)  21 (52.5)  8 (20.0)  2 (5.0)  5 (12.5)  9 (22.5) | 0.72  0.69  0.32  0.03  0.43  0.58  0.49  0.48  0.01  0.001  0.26  0.002  0.38  0.45  0.51  0.30  0.58  0.34  0.51 |
| **Family History**  Obesity  Food Allergies  Diabetes  Heart Disease  Cancer  HTN  Dyslipidemia | 59 (81.9)  17 (23.6)  59 (81.9)  37 (51.4)  30 (41.7)  59 (81.9)  25 (34.7) | 25 (78.1)  5 (15.6)  26 (81.3)  12 (37.5)  10 (31.3)  26 (81.3)  8 (25.0) | 34 (85.0)  12 (30.0)  33 (82.5)  25 (62.5)  20 (50.0)  33 (82.5)  17 (42.5) | 0.33  0.13  0.56  0.03  0.09  0.56  0.09 |
| **Smoking Status**  Never  Occasional  Frequent  If yes, duration (years)  2  4  5  7  10  30  How many cigarettes/day  1  3  4  5  10  20  40 | 56 (77.8)  5 (6.9)  11 (15.3)  1 (6.3)  3 (18.8)  4 (25.0)  1 (6.3)  5 (31.3)  2 (12.5)  1 (6.3)  1 (6.3  3 (18.8)  1 (6.3)  1 (6.3)  6 (8.3)  3 (18.8) | 22 (68.8)  2 (6.3)  8 (25.0)  2 (20.0)  3 (30.0)  1 (10.0)  2 (20.0)  2 (20.0)  1 (10.0)  2 (20.0)  --  --  5 (50.0)  2 (20.0) | 34 (85.0)  3 (7.5)  3 (7.5)  1 (16.7)  1(16.7)  1(16.7)  --  3 (50.0)  1 (16.7)  --  1 (16.7)  1 (16.7)  1 (16.7)  1 (16.7) | 0.12 |
| **Physical Activity**  If yes, amount per day  20 minutes  30 minutes  60 minutes  >60 minutes | 19 (26.4)  3 (15.8)  7 (36.8)  6 (31.6)  3 (15.8) | 8 (25.0)  2 (25.0)  4 (50.0)  --  2 (25.0) | 11 (27.5)  1 (9.1)  3 (27.3)  6 (54.5)  1 (9.1) | 0.52 |
